# Subpopulations of dermal skin fibroblasts secrete distinct extracellular matrix: implications for using skin substitutes in the clinic†

**DOI:** 10.1111/bjd.16255

**Published:** 2018-05-16

**Authors:** M. Ghetti, H. Topouzi, G. Theocharidis, V. Papa, G. Williams, E. Bondioli, G. Cenacchi, J.T. Connelly, C.A. Higgins

**Affiliations:** ^1^ Biomedical and Neuromotor Sciences Department University of Bologna Bologna Italy; ^2^ Burns Centre and Emilia Romagna Regional Skin Bank Cesena Italy; ^3^ Department of Bioengineering Imperial College London London U.K.; ^4^ Centre for Cell Biology and Cutaneous Research Barts and the London School of Medicine and Dentistry Queen Mary University London London U.K.; ^5^ Farjo Hair Institute Manchester U.K.

## Abstract

**Background:**

While several commercial dermoepidermal scaffolds can promote wound healing of the skin, the achievement of complete skin regeneration still represents a major challenge.

**Objectives:**

To perform biological characterization of self‐assembled extracellular matrices (ECMs) from three different subpopulations of fibroblasts found in human skin: papillary fibroblasts (Pfi), reticular fibroblasts (Rfi) and dermal papilla fibroblasts (DPfi).

**Methods:**

Fibroblast subpopulations were cultured with ascorbic acid to promote cell‐assembled matrix production for 10 days. Subsequently, cells were removed and the remaining matrices characterized. Additionally, in another experiment, keratinocytes were seeded on the top of cell‐depleted ECMs to generate epidermal‐only skin constructs.

**Results:**

We found that the ECM self‐assembled by Pfi exhibited randomly oriented fibres associated with the highest interfibrillar space, reflecting ECM characteristics that are physiologically present within the papillary dermis. Mass spectrometry followed by validation with immunofluorescence analysis showed that thrombospondin 1 is preferentially expressed within the DPfi‐derived matrix. Moreover, we observed that epidermal constructs grown on DPfi or Pfi matrices exhibited normal basement membrane formation, whereas Rfi matrices were unable to support membrane formation.

**Conclusions:**

We argue that inspiration can be taken from these different ECMs, to improve the design of therapeutic biomaterials in skin engineering applications.

## Abstract 2

**What's already known about this topic?**

- There are several types of skin fibroblasts within the dermis that can be defined by their spatial location: papillary fibroblasts (Pfi), reticular fibroblasts (Rfi) and dermal papilla fibroblasts (DPfi).
- Extracellular matrix (ECM) composition is distinct with regard to composition and architecture within the papillary, reticular and hair follicle dermis *in vivo*.
- When skin is injured, dermal replacement substitutes used for tissue repair do not reflect the heterogeneity observed within the skin dermis.

**What does this study add?**

- Self‐assembled ECMs from different subpopulations of skin fibroblasts can be generated *in vitro*.
- Cell‐assembled ECMs made *in vitro* from Pfi, Rfi and DPfi reflect dermal heterogeneity seen *in vivo* and are morphologically, functionally and compositionally distinct from one another.
- Inspiration should be taken from cell‐assembled ECMs from distinct fibroblast subpopulations, to improve the design of therapeutic biomaterials in skin engineering applications.

**What is the translational message?**

- Cell‐assembled ECMs from DPfi and Pfi, but not Rfi, can support formation of a basement membrane in adjacent keratinocytes in vitro.
- Inspiration should be taken from cell‐assembled ECMs from distinct fibroblast subpopulations, to improve the design of therapeutic biomaterials in skin engineering applications.

**Linked Comment:** https://doi.org/10.1111/bjd.16773.

https://doi.org/10.1111/bjd.16946 available online

https://goo.gl/Uqv3dl

Skin is a multilayered structure comprised of an underlying supporting dermis and functional epithelium at the skin surface. This is oversimplified, as within each layer there are multiple cells with different functions that contribute to tissue homeostasis. Within the epidermis, keratinocytes are the most abundant cell type; these differentiate and stratify towards the skin surface, equipping skin with its barrier function.^1^ Within the skin dermis the most abundant cell type is fibroblasts, the primary role of which is to secrete components of the extracellular matrix (ECM) for structural support.^2^ Largely unconsidered, there are several types of skin fibroblasts within the dermis, which can be defined by their spatial location,^3^ and exist as morphologically and functionally heterogeneous subpopulations.^4^, ^5^, ^6^ For example, the fibroblast cells within the dermal layer adjacent to the basement membrane and epidermis known as the papillary dermis are called papillary fibroblasts (Pfi). These are distinct from those residing within the lower reticular dermis, which are termed reticular fibroblasts (Rfi).^4^, ^6^ There are also fibroblasts with a fibrogenic tendency, which are thought to be responsible for scar deposition after wounding.^5^ However these are distributed in a speckled pattern throughout the reticular and papillary dermis, and are therefore harder to define by spatial location.^5^ If we label cells according to their location, rather than their behaviour, then Pfi reside adjacent to the basement membrane, where they direct growth and differentiation of epidermal keratinocytes.^7^ Comparatively, the deeper Rfi produce the bulk of the dermal ECM and are responsible for the first wave of dermal repair following a full‐thickness wound.^6^ In hairy skin there are also fibroblasts associated with the hair follicle, located in the dermal papilla and the connective tissue sheath.^3^, ^8^, ^9^, ^10^, ^11^, ^12^ Dermal papilla fibroblasts (DPfi) have specialized signalling properties required for hair follicle morphogenesis and coordination of hair growth.^13^ In the hair follicle a specialized basement membrane, termed a glassy membrane, separates the dermal papilla from the surrounding epithelial matrix.^14^ In addition, the human dermal papilla is rich in interstitial collagens, such as type I and type III, in addition to fibrillar matrix proteins such as fibronectin 1 (FN1), glycoproteins such as thrombospondin 1 (THBS1),^14^, ^15^, ^16^, ^17^ and proteoglycans such as versican.^18^ Various studies have also demonstrated that ECMs produced by Pfi and Rfi within the interfollicular dermis are distinct with regard to their composition and architecture.^4^, ^12^, ^19^, ^20^ Pfi in the upper dermis secrete ECM, which is constituted of thin, poorly organized collagen fibre bundles, whereas thick, well‐organized collagen bundles are characteristic within the lower dermis, which is produced by resident Rfi.^4^ The papillary dermis also has a higher ratio of collagen type III to type I, higher levels of the dermatan sulfate proteoglycan decorin, yet lower levels of the chondroitin sulfate proteoglycan versican than the reticular dermis.^4^, ^20^, ^21^, ^22^ Not only do differences between fibroblast subtypes control how they behave and interact with surrounding cell types, but the ECM secreted by fibroblasts in distinct subanatomical locations also has a key role in the regulation of these interactions. When fibroblasts are isolated from the skin and grown in culture they retain distinct transcriptional signatures despite being removed from environmental cues.^23^ Concomitantly with this, Pfi have been shown to synthesize more decorin than Rfi in culture, reflecting the expression pattern of decorin in the skin *in vivo*.^20^

It is important to understand fibroblast behaviour within the skin, especially in the context of wound healing, if we want to successfully modulate this process. After injury in the skin, there are coordinated processes that lead to re‐epithelialization and establishment of a new functional barrier; however, it is the repair and ECM deposition within the skin dermis that results in scar formation. Specifically, fibroblasts synthesize highly aligned and bulky ECM fibres after injury, and a consequence of this is that complete skin regeneration cannot be achieved.^24^, ^25^

After injury, or in the case of a chronic skin wound, there are a number of biological products available on the market that promote healing which have been developed by combining primary skin cells with biomaterials.^26^, ^27^, ^28^, ^29^ These skin substitutes are biologically active through the release of growth factors and cytokines that aid the recruitment and adhesion of host cells.^30^, ^31^, ^32^ There are a small number of bioengineered skin products that replace both the epidermal and dermal layers of the skin; one such example is Apligraf^®^, which is composed of neonate‐derived fibroblasts cultured in a bovine collagen matrix, over which neonate‐derived keratinocytes are seeded to produce a stratified epidermis. In recent years, a group of biological scaffolds comprised of ECMs that are used solely as a dermal replacement have received increasing interest.^33^, ^34^, ^35^, ^36^ One of these is Integra^®^, a dermal regeneration template composed of a layer of bovine tendon collagen type I matrix and shark chondroitine‐6‐sulfate juxtaposed against a silicone layer that acts as a temporary pseudo‐epidermis.^37^, ^38^ Despite the success of Integra, there are some limitations. Two operations are necessary, and there are risks of infection under the silicone layer and a risk that the silicone will become detached.^39^ An alternative to this is MatriDerm^®^, which is an engineered dermal template specially developed to provide a one‐step grafting procedure. MatriDerm is a scaffold consisting of a native bovine type I, III and V collagen fibre template incorporating elastin hydrolysate that is converted into native host collagen within weeks of application.^40^ In addition to engineered scaffolds, decellularized skin dermis from cadaveric donors is now becoming more popular as a scaffold for use after injury where there is extensive skin loss.^41^ The common feature of all these decellularized dermal scaffolds is that the native collagen fibres guide fibroblasts and possibly other cells toward dermal regeneration, whereas the presence of elastin in the collagen matrix diminishes the formation of granulation tissue in the early phase of wound healing.^42^ As a result, a high‐quality neodermis with randomly organized collagen bundles can be regenerated. These decellularized dermal scaffolds are commonly taken from reticular dermis rather than papillary dermis. Reticular dermis is much thicker than papillary dermis, and therefore provides a greater area for cell infiltration. In addition, its relatively low cellularity in comparison with the papillary dermis means it is easy to decellularize. Lastly, the reticular dermis shows a strong mechanical resistance when compared with papillary dermis, making it easy to handle.^43^ Despite these advantages, decellularized reticular dermis does not have a basal membrane and this feature can make re‐epithelialization of grafts by host keratinocytes cells more difficult.^44^

As highlighted above, one problem with dermal replacement scaffolds is they do not reflect the heterogeneity observed within the skin dermis, where different fibroblasts subtypes, including the ones described herein, have divergent functions.^1^ Furthermore, with decellularized scaffolds there can be issues associated with inflammation, and these foreign body reactions have led scientists to investigate new strategies based on *in vitro* organogenesis approaches.^45^ A therapeutic strategy such as this requires cells to first be obtained from their native tissue, then kept in long‐term culture with appropriate growth factors to produce a tissue substitute rich in ECM.^46^, ^47^, ^48^, ^49^ In this respect, ECM produced by cells via a self‐assembly approach has been shown to act as a key cell adhesion site and a mechanically strong scaffold‐free support for tissue engineering.^50^, ^51^, ^52^ Finally, cell‐derived ECM of mesenchymal cells from anatomically distinct sites, such as the bone marrow and adipose tissue, can be created *in vitro* and reflect the distinct ECM found in these different sites.^53^

Based on the above observations, in this study we decided to investigate cell‐derived ECMs as potential scaffolds for use in skin engineering. We had two main objectives: the first was to generate and biologically characterize ECMs produced by fibroblast subpopulations found both within the hair follicle dermis, and the papillary and reticular dermis of human skin. We refer to these fibroblasts using names based on their subanatomical origin. Our second aim was to evaluate how ECMs from fibroblasts in different dermal locations, which are from areas juxtaposed to basement membrane or devoid of basement membrane, can interact with and instruct basement membrane formation in epithelial only skin constructs.

## Materials and methods

### Human tissue samples and cell culture

Scalp skin tissues were taken from healthy donors, using Imperial College Research Ethics Committee‐approved consent forms, and used for the isolation of epidermal keratinocytes and dermal subpopulations. Specifically, Pfi and Rfi were obtained from the upper and lower regions of interfollicular dermal tissue, respectively. To isolate cells, the adipose tissue was first cut off the scalp tissue using a scalpel. The tissue was then laid flat within a Petri dish containing phosphate‐buffered saline (PBS) then, using a stereo‐dissection microscope to view the tissue, a scalpel was used to transect the dermis 100–200 μm below the epidermis. The piece of tissue containing epidermis and papillary dermis was used to isolate Pfi, whereas the deeper dermis was used for isolation of Rfi. These pieces of superficial (papillary) and deep (reticular) tissue were minced, then placed in culture in different 35‐mm dishes in Dulbecco's Modified Eagle's Medium (DMEM) containing GlutaMAX (Gibco, Invitrogen, Carlsbad, CA, U.S.A.) supplemented with 20% fetal bovine serum (FBS) (Gibco, Invitrogen) and 1% antibiotics–antimycotics (Gibco, Invitrogen). Tissue was maintained at 37 °C in a humidified atmosphere of 5% CO_2_/95% air for 10 days, during which time Pfi and Rfi migrated from their respective dermal explants. After migration of fibroblasts outward from the explants, cells were amplified in culture in DMEM GlutaMAX, 10% FBS and 1% antibiotics at 37 °C in a humidified atmosphere of 5% CO_2_/95% air, and passaged until passage 3 (P3), at which point they were used to establish cell‐derived ECMs.

Intact dermal papillae were also isolated from the same piece of scalp skin using a microdissection approach to obtain DPfi cultures from the same donors as the Pfi and Rfi.^54^ Follicles were transected just above the level of the dermal papilla to isolate end bulbs, which were inverted using 27‐gauge needles to remove the matrix and expose the dermal papilla. Papillae were then separated from the follicle by cutting through their stalk. For culture, eight papillae were transferred to 35‐mm dishes containing 20% FBS in DMEM GlutaMAX, with 1% antibiotics–antimycotics. Cells migrated from the papillae and when the dish was approaching confluence, DPfi were passaged at a 1 : 2 ratio using 0·5% trypsin–ethylenediaminetetraacetic acid (EDTA; Gibco, Invitrogen) for detachment. After the initial 2 weeks of culture, cells were grown in DMEM GlutaMAX, 10% FBS and 1% antibiotics. After passaging, DPfi were cultured in the same manner as Pfi and Rfi. Matched sets of cells, from three different male donors, were used for ECM generation and subsequent analysis.

To assess proliferation characteristics of fibroblast subtypes, cells were seeded at 6000 cells per cm^2^. After 24 and 168 h, 100 μl alamarBlue^®^ reagent (Invitrogen) was added directly to cells in 1 mL culture medium. The cells were incubated for 3 h at 37 °C, protected from direct sunlight. After 3 h, 100 μL aliquots were taken in duplicate and their absorbance was measured at 570 nm, using 600 nm as a reference wavelength. The fluorescence intensity is proportional to cell number.

For keratinocyte isolation, skin samples were washed briefly in PBS containing 2% antibiotics–antimycotics, then incubated in Dispase (Stem Cell Technologies, Vancouver, Canada) overnight at 4 °C. The epidermis and dermis were then separated from each other with fine forceps. Next, the epidermal layer was minced with scissors and incubated in 0·5% trypsin–EDTA at 37 °C for 30 min. Digested tissue was filtered through a 70‐μm cell strainer, then cells were pelleted and resuspended in EpiLife medium with 1% antibiotics and EpiLife Defined Growth Supplement (Gibco, Invitrogen). Cells were grown at 37 °C in a humidified atmosphere of 5% CO_2_/95% air and used for epithelial‐only skin construct generation at P2.

### Self‐assembly approach for matrix generation

To generate ECMs from dermal fibroblast subpopulations we followed previously described protocols.^55^ Sterile 13‐mm glass coverslips in a 24‐well plate were coated with 0·2% sterile gelatin (gelatin type B; Sigma Aldrich, St Louis, MO, U.S.A.) for 60 min at 37°C. They were washed three times with PBS, cross‐linked with 1% sterile glutaraldehyde (Sigma Aldrich) for 30 min at room temperature (RT) and again washed three times with PBS. Crosslinking was quenched with 1 mol L^−1^ sterile glycine in PBS for 20 min at RT, followed by three more washes in PBS. Coverslips were then incubated in growth medium (DMEM GlutaMAX, 10% FBS, 1% antibiotics) for 30 min at 37°C. Finally, coverslips were washed three more times with PBS, then used immediately. The three different subpopulations of fibroblasts (DPfi, Pfi, Rfi) were plated onto coverslips in 24‐well plates. A total of 65 000 cells (34 000 cells cm^−2^) were seeded into each well in DMEM GlutaMAX, 10% FBS and 1% antibiotics, and cultured overnight at 37°C, 5% CO_2_/95% air to achieve a confluent lawn of fibroblasts. The next day, growth medium supplemented with 50 μg mL^−1^ ascorbic acid (Sigma Aldrich) was added to each culture to promote self‐assembly of ECM from each cell type. Medium was replaced with fresh medium every 2 days for a total of 10 days. After 10 days of culture in ascorbic acid‐supplemented medium, cells were removed. Medium was aspirated and cells were washed once with PBS before prewarmed extraction buffer (20 mmol L^−1^ NH_4_OH, 0·5% Triton X‐100 in PBS) was added and left for 4 min to allow cell lysis. Half the buffer was carefully removed and PBS was added. The same step was repeated until no intact cells were visible. The DNA residue was digested with 10 μg mL^−1^ DNase I (Roche, Indianapolis, IN, U.S.A.) in PBS for 30 min at 37°C in 5% CO_2_, followed by two washes with PBS. Matrices were either used immediately or stored at 4 °C in PBS with 1% antibiotics for up to 4 weeks.

### Immunofluorescence staining and analysis of cell‐assembled extracellular matrices

We performed immunofluorescence of specific proteins in the self‐assembled matrices using antibodies against individual ECM components. Matrices were fixed using 4% paraformaldehyde (PFA) for 20 min, followed by wash and blocking steps with 5% goat serum (Vector Laboratories, Burlingame, CA, U.S.A.) in PBS for 30 min. Primary antibodies were diluted in PBS and placed on matrices overnight at 4°C (Table 1). Secondary antibodies were used for 1 h at RT (Table 1). Finally, the matrices were washed three times in PBS and mounted on glass slides with Vectashield mounting medium (Vector Laboratories). After staining, ECMs were imaged using a Zeiss LSM‐510 inverted confocal microscope (Carl Zeiss, Jena, Germany). For each FN1‐stained sample, three random Z‐stack images of matrices were acquired; these were used for the evaluation of alignment and fibre measurements. The thickness of the ECMs was determined by taking Z‐stack images on the confocal microscope and subtracting the distance between the top and the bottom of assembled FN1 matrix. The images were subsequently processed with the ImageJ (National Institutes of Health, Bethesda, MD, U.S.A.) and Fiji (http://fiji.sc) programs. Each Z‐stack set of images was converted to a single image by using the maximum projection function. The ‘Dimensionality’ plug‐in was applied to calculate the orientation distribution of fibres, whereas the diameter of fibres and interfibrillar spaces were assessed with the ‘BoneJ’ plug‐in.^56^ All graphs were generated using GraphPad Prism version 6·01 (GraphPad Inc., La Jolla, CA, U.S.A.). Data are shown as the mean; error bars (±) are the SD of the mean. A one‐way anova followed by Bonferroni's correction was performed for experiments, with *P* < 0·05 considered significant.

**Table 1:** Primary and secondary antibodies used in this study

Antigen	Source	Catalogue number	Species (raised in)	Dilution used
Fibronectin	Sigma‐Aldrich^a^	F3648‐5mL	Rabbit	1 : 500
Collagen 1	Abcam^b^	ab90395	Mouse	1 : 300
Tenascin C	Abcam^b^	ab6393	Mouse	1 : 300
Collagen 6	Abcam^b^	ab6588	Rabbit	1 : 500
Thrombospondin	Abcam^b^	ab1823	Mouse	1 : 50
Collagen 7	Abcam^b^	ab93350	Rabbit	1 : 500
Collagen 4	Abcam^b^	ab6583	Mouse	1 : 50
Antimouse–Alexa Fluor 488	Molecular Probes^c^	A‐11001	Goat	1 : 200
Antirabbit–Alexa Fluor 546	Molecular Probes^c^	A‐11010	Goat	1 : 200

^a^St Louis, MO, U.S.A.; ^b^Cambridge, U.K.; ^c^Eugene, OR, U.S.A.

### Generation of epidermal‐only skin constructs

For this experiment we used fibroblasts and keratinocytes isolated as described above. To start, we placed several cell inserts (Millicell‐24 Cell Culture Insert Plate, polycarbonate, 0·4 μm; Millipore, Burlington, MA, U.S.A.) into a 60‐mm cell culture dish, and coated them using the same method for coverslip coating as described earlier. We seeded each fibroblast subpopulation at a density of 30 000 cells per insert in DMEM GlutaMAX, 10% FBS, 1% antibiotics and allowed the fibroblasts to produce ECMs for 10 days in ascorbic acid‐supplemented medium on the inside of each insert. After removing fibroblasts from these matrices, we seeded 250 000 keratinocytes inside each insert on the top of the cell‐depleted ECMs in Cnt Prime Medium (CellnTec, Bern, Switzerland). After 2 days, we removed the Cnt Prime Medium from the inserts and added 3D Barrier Medium (CellnTec) both inside and outside the inserts. The next day all 3D Barrier Medium was removed and replaced with fresh 3D Barrier Medium on the outside of the inserts only. This enabled establishment of an air–liquid interface, which is required for epithelial stratification. Finally, we kept the cells for 14 days in culture at the air–liquid interface, prior to analysis as described earlier.

### Skin construct viability, immunofluorescence staining and transmission electron microscopy

We used an alamarBlue assay to assess cell viability of epidermal skin constructs after 14 days of culture. Resazurin, the active ingredient of alamarBlue, undergoes reduction on exposing cells to resorufin, a compound that is red in colour and highly fluorescent. Viable cells continuously convert resazurin to resorufin, increasing the overall fluorescence and colour of the medium surrounding cells. alamarBlue dye, at a concentration of 10% in PBS, was added to each skin construct and incubated for 4 h at 37 °C in 5% CO_2_/95% air. Subsequently, 100 μL medium was transferred to a fresh 96‐well plate and absorbance was read at 570 nm. Data are expressed as absorbance value units.

Alternatively, after the growth period of 14 days at the air–liquid interface, samples were snap‐frozen in OCT mounting medium and stored at –80 °C until processing. Frozen samples were sectioned into 7‐μm slides using a cryostat. For staining, these were fixed with either 4% PFA in PBS for 10 min at RT, or 100% methanol for 7 min at –20 °C. After fixation, the samples were rinsed three times with PBS and pretreated for 30 min with PBS containing 5% goat serum, followed by incubation overnight at 4°C with primary antibodies (Table 1). The sections were thoroughly rinsed with PBS and then incubated with secondary antibodies for 1 h at RT. Coverslips were mounted using Vectashield containing DAPI (Vector Laboratories), which subsequently labels nuclei. Confocal microscopy (Zeiss LSM‐510 inverted) was used to visualize and capture immunostained cells. As a positive control, human skin was stained using the same antibodies.

For transmission electron microscopy (TEM), 14‐day‐old constructs were fixed in 2·5% glutaraldehyde in 0·1 M cacodylate buffer at pH 7·2–7·4, washed in 0·1 mol L^−1^ cacodylate buffer at pH 7·2, postfixed in 1% OsO_4_ in 0·1 mol L^−1^ cacodylate buffer at pH 7·2, dehydrated in graded ethanol and embedded in Araldite (Serva, Heidelberg, Germany). Ultrathin sections were counterstained with uranyl acetate and lead citrate, and examined under a Zeiss EM109 transmission electron microscope to evaluate basement membrane formation within the constructs.

### Extracellular matrix samples directly analysed by mass spectrometry

The protocol we used for mass spectrometry (MS) was described previously by Pflieger *et al*.^57^ After cell elimination, ECM proteins were directly proteolysed in each well using 2·5 μg trypsin (Gibco, Invitrogen) in 250 μL 30 mmol L^−1^ Tris (pH 8·0) at 37°C overnight. Predigested matrix proteins were scraped off from the wells using a cell scraper and collected into 1·5 mL microfuge tubes. The samples were then reduced by the addition of dithiothreitol (final concentration 10 mmol L^−1^) and incubated for 30 min at 56 °C. After reduction, iodoacetamide (final concentration 55 mmol L^−1^) was added to prevent the disulfide bonds re‐forming and thus keep the protein unfolded, and samples were incubated for 30 min at RT in the dark. Finally, the samples were further digested by trypsin at 37 °C overnight before terminating the digest with the addition of 10% trifluoroacetic acid (final concentration 0·5%).

Samples were analysed by liquid chromatography MS using a nanoACQUITY UPLC™ system (Waters MS Technologies, Manchester, U.K.). One microlitre (1–3 μg protein digest) of sample was injected onto each trapping column (C18, 180 μm × 20 mm; Waters) using partial loop injection, for 1 min at a flow rate of 15 μL min^−1^ with 0·1% (v/v) formic acid. Samples were resolved on an analytical column (nanoACQUITY UPLC™ M‐class HSS T3 75 μm × 150 mm 1·8 μm column; Waters) using a gradient of 97% A [0·1% (v/v) formic acid], 3% B [99·9% acetonitrile 0·1% (v/v) formic acid] to 60% A, 40% B over 36 min at a flow rate of 300 nL min^−1^. The nanoACQUITY UPLC was coupled to a Synapt ™ G2 mass spectrometer (Waters) and data were acquired using a MS^E^ program with 1‐s scan times and a collision energy ramp of 15–40 eV for elevated energy scans. The mass spectrometer was calibrated before use and throughout the analytical run at 1‐min intervals using the NanoLockSpray™ (Waters) source with Glu‐fibrinopeptide.

To analyse quantitatively the MS results, peptide identification was performed using the ProteinLynx Global SERVER™ (PLGS) v3·0·3 (Waters). The data were processed using a low‐energy threshold of 250. A fixed carbamidomethyl modification for cysteine was specified. The search thresholds used were as follows: minimum fragment ion matches per peptide 3; minimum fragment ion matches per protein 7; minimum peptides per protein 1; a false‐positive value of 4. The maximum protein mass identified was 50 kDa. Data were searched against the most recent UniProtKB/Swiss‐Prot human database entry.^58^ For normalization, each sample had a known trypsin concentration, which was used as an internal standard. All protein hits were identified with a confidence of more than 95%. The PLGS protein score was based on the probability that the observed match between the experimental data and the PLGS database was not a random event, thus minimizing potential random hits. Furthermore, if two or more proteins shared an identical peptide, but the peptide in question was regulated differently, then the peptide was not included in the analysis. Peptide intensity was determined as the sum of the peptide ions for all unique peptides for each protein. A total peptide score was taken as the integrated total of all normalized peptide intensities, for all identified ECM proteins.^59^ Analysis was performed on matrices derived using cells from two different patients.

## Results

### Fibroblast subpopulations produced self‐assembled extracellular matrices with different morphology and architectural structure

**Figure 1:**
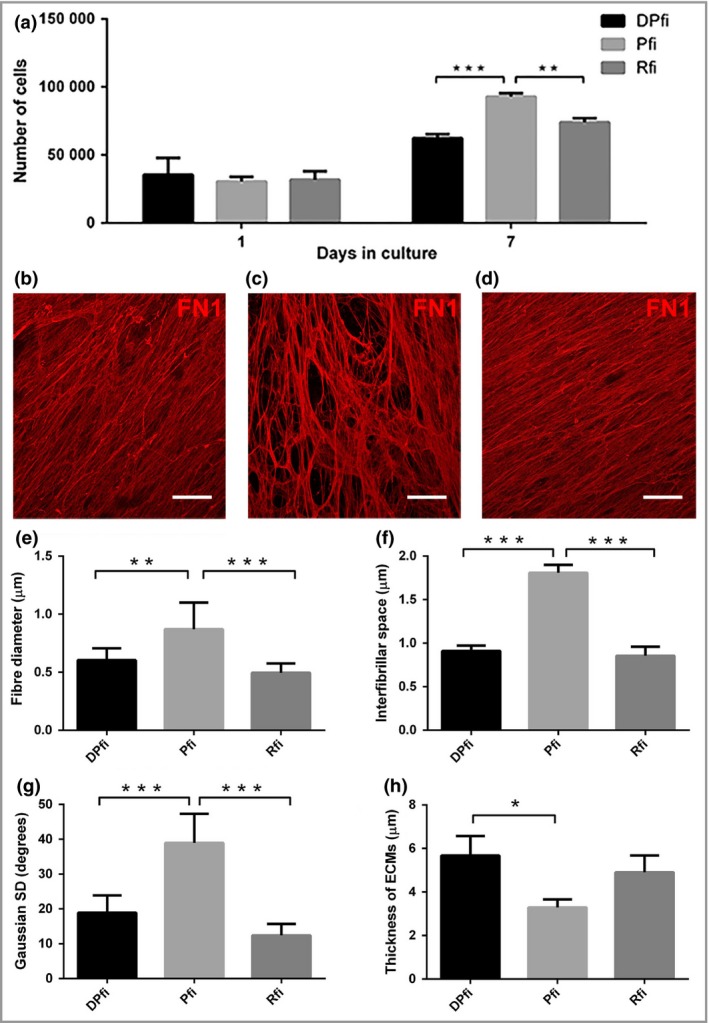
Morphological characterization of cell‐assembled extracellular matrices (ECMs). (a) Cell numbers, 1 and 7 days after seeding cells at equivalent densities, highlight fibroblast subtype‐specific differences. Representative immunofluorescence images of fibronectin 1 (FN1)‐stained ECMs generated by (b) dermal papilla fibroblasts (DPfi), (c) papillary fibroblasts (Pfi) or (d) reticular fibroblasts (Rfi) after 10 days in culture. Corresponding graphs determined from analysis of nine representative images show quantification of fibre diameter for each (e) cell‐assembled ECM, (f) interfibrillar space, (g) Gaussian SD of fibre orientation and (h) total ECM thickness. Data are mean ± SD (*n* = 3). *P < 0·05, **P < 0·01, ***P < 0·001. Scale bars in (a–c) are 50 μm.

We first generated cell‐assembled matrices from each of the three fibroblast subpopulations: DPfi, Pfi and Rfi. These were seeded to achieve full confluency immediately and switched to matrix assembly media at 24 h, to ensure differential proliferation profiles had no effect on matrix production (Fig. 1a). Using confocal microscopy images of FN1 coupled with image analysis algorithms (Fig. 1b–d), we found that the architecture of the self‐assembled ECMs varied between each of the three fibroblast subpopulations used. Specifically, Pfi generated matrices with significantly thicker fibres than DPfi and Rfi, which were associated with the highest amount of interfibrillar space between each fibre (Fig. 1e, f). The Pfi‐derived ECM fibres were also significantly anisotropic compared with both Rfi‐ and DPfi‐derived matrices (Fig. 1g). This bears resemblance to the papillary dermis, which is disorganized in comparison with the reticular dermis.^4^ Lastly, when we assessed matrix thickness, the DPfi deposited significantly more matrix than the Pfi (Fig. 1h).

### Fibroblast subpopulations differentially support basement membrane formation

**Figure 2:**
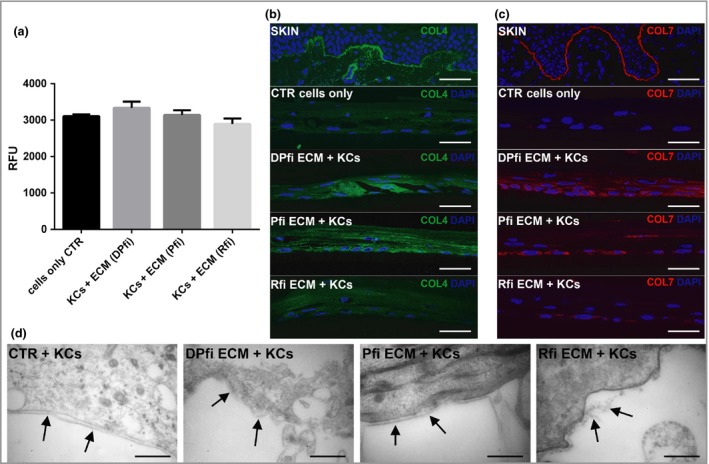
Epithelial‐only skin constructs grown on cell‐assembled extracellular matrices (ECMs). (a) Viability assay of cultured keratinocytes (KCs) on different fibroblast‐derived matrices (mean ± SD). Immunofluorescence images of (b) collagen IV (COL4) and (c) collage VII (COL7) in entire skin and epithelial‐only constructs cultured on coating matrix, or dermal papilla fibroblast (DPfi), papillary fibroblast (Pfi) and reticular fibroblast (Rfi)‐derived ECM. (d) Ultrastructural analysis of the basement membrane (arrows) in the epithelial‐only skin constructs (*n* = 3). Scale bars for (b, c) are 50 μm. Scale bars for (d) are 400 nm. RFU, relative fluorescence unit; CTR, control; DAPI, 4’,6‐diamidino‐2‐phenylindole.

After determining that ECMs could be successfully generated from each fibroblast subtype, we decided to employ a protocol to generate epithelial‐only skin constructs, replacing the collagen coating that is usually used with ECM assembled from each cell type. Using alamarBlue to assess keratinocyte viability, we found no significant statistical differences between epithelial‐only constructs grown on different self‐assembled ECMs compared with the control (Fig. 2a). Next, we specifically wanted to assess basement membrane formation, as our fibroblast subpopulations were derived from subanatomical locations subjacent to, and devoid of, basement membrane. We used antibodies against collagen IV (COL4) and collagen VII (COL7) to assess basement membrane characteristics. COL4, which is deposited by both fibroblasts and keratinocytes,^60^ was observed only in keratinocytes grown on Pfi ECM and DPfi ECM (Fig. 2), but not in keratinocytes supported by Rfi ECM or control constructs (without matrix). COL7, the production of which in keratinocytes is stimulated by fibroblasts,^61^ was also found in keratinocytes in both DPfi and Pfi matrix‐supported constructs. However, COL7 was not expressed in the control group or in the Rfi ECM supported constructs (Fig. 2c). In addition to immunofluorescence, we used TEM to assess basement membrane formation in our epithelial‐only skin constructs (Fig. 2d). Basement membranes were detected in the control group, despite not identifying COL4 or COL7 by immunofluorescence. We also detected basement membrane in constructs supported by Pfi and DPfi matrices but not Rfi‐derived matrices. The skin constructs grown on Pfi matrices had a very clear and continuous basal lamina structure compared with the control. In contrast, the skin constructs supported by DPfi‐derived ECM had a very thick basement membrane; however, this was not continuous across the entire construct.

### Mass spectrometry revealed different matrix compositions

Given the different fibre morphologies in our self‐assembled ECMS from different fibroblast subpopulations, and a divergent ability to support establishment of a basement membrane, we wanted to determine whether there were also differences in matrix composition. To do this, we performed MS analysis of digested matrices devoid of cells.

Our analysis identified a number of different proteins with the maximum protein mass of 50 kDa. In total, 12 ECM proteins were identified that passed the 95% accuracy threshold within the DPfi, Pfi and Rfi cell‐assembled ECMs (Table 2). Based on the total peptide ion count, DPfi produced significantly more ECM compared with either the Rfi or Pfi over a 10‐day time frame. All identified proteins were interstitial ECM rather than basement membrane ECM. Specifically, FN1, which was used for image analysis, was produced equally by all three different fibroblast subpopulations. Surprisingly, collagen I (COL1), which is generally thought to be expressed throughout the dermis, was not found in either of the Pfi ECM samples, even if we considered peptides that did not pass the accuracy cut‐off threshold.

**Table 2:** Mass spectrometry data of extracellular matrices (ECM) for each fibroblast subtype

Protein symbol	Protein ID	DPfi ECM			Pfi ECM			Rfi ECM			ECM type
		PLGS score^a^	Coverage (%)^b^	Peptide intensity sum^c^	PLGS score^a^	Coverage (%)^b^	Peptide intensity sum	PLGS score^a^	Coverage (%)^b^	Peptide intensity sum^c^	
FN1	P02751	13937	49	9138740	4245	44	4349942	4511	47	6198948	Adhesive glycoprotein
COL1A1	P02452	241	25	319566	–	–	–	–	–	–	Fibrous
COL1A2	P08123	1018	15	398538	–	–	–	89	14	26986	Fibrous
COL6A1	P12109	5787	42	1399723	208	18	79824	259	26	142226	Fibrous
COL6A2	P12110	2448	28	721444	–	–	–	–	–	–	Fibrous
COL6A3	P12111	2561	34	3888636	226	23	263054	268	25	683339	Fibrous
TNC	P24821	1931	30	1579325	549	27	647935	681	30	885657	Adhesive glycoprotein
THBS1	P07996	1014	31	575557	–	–	–	129	21	106958	Adhesive glycoprotein
FBLN2	P98095	431	23	171414	129	17	61035	238	24	217243	Ca^2+^ binding glycoprotein
VTN	P04004	403	16	44674	95	18	12991	–	–	–	Adhesive glycoprotein
FBN1	P35555	–	–	–	–	–	–	118	17	136846	Adhesive glycoprotein
EMIL1	Q9Y6C2	–	–	–	–	–	–	95	9	21431	Adhesive glycoprotein
Total peptide intensity^d^		18237614			5414781			8392648			

Listed are the protein symbol and accession number (ID) from Swiss‐Prot for all the ECM proteins detected. ^a^A statistical measure of peptide assignment accuracy. ^b^Percentage of protein sequence covered by identified peptides for each protein. ^c^Sum of the peptide ion intensities for each protein, normalized against a trypsin internal control. ^d^Sum of the peptide ions for all peptides identified, for each ECM protein. Values are the average of matrices from *n* = 2. DPfi, dermal papilla fibroblasts; Pfi, papillary fibroblasts; Rfi, reticular fibroblasts; PLGS, ProteinLynx Global SERVER; FN1, fibronectin 1; COL1A1, α1 chain collagen I; COL1A2, α2 chain collagen II; COL6A1, α1 chain collagen VI; COL6A2, α2 chain collagen VI; COL6A3, α3 chain collagen VI; TNC, tenascin; THBS1, thrombospondin 1; FBLN2, fibulin 2; VTN, vitronectin; FBN1, fibrillin 1; EMIL1, emilin 1.

**Figure 3:**
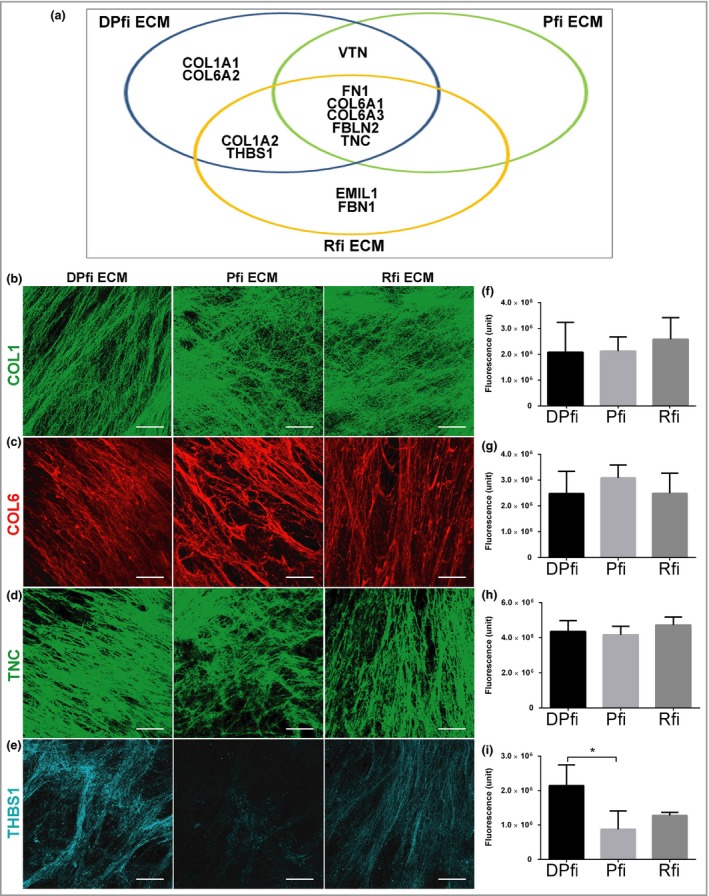
Extracellular matrix (ECM) composition. (a) Venn diagram showing distribution of 12 ECM components in matrices assembled from dermal papilla fibroblast (DPfi), papillary fibroblast (Pfi) and reticular fibroblast (Rfi). Representative immunofluorescence images of (b) collagen I (COL1), (c) collagen VI (COL6), (d) tenascin (TNC) and (e) thrombospondin 1 (THBS1) in ECMs generated by DPfi, Pfi or Rfi cells after 10 days in ascorbic acid‐supplemented medium. (f–i) Quantification of immunofluorescence is plotted in graphs as the mean ± SD (*n* = 3). *P < 0·05. Scale bars in (b–e) are 50 μm. COL1A1, α1 chain collagen I; COL6A2, α2 chain collagen VI; COL1A2, α2 chain collagen II; VTN, vitronectin; FN1, fibronectin 1; COL6A1, α1 chain collagen VI; COL6A3, α3 chain collagen VI; FBLN2, fibulin 2.

Interstitial ECM is usually classified into fibrous ECMs (collagens and elastin), proteoglycans or ‘other’. Intriguingly, our analysis identified collagens and proteins that fell into the ‘other’ category, but no proteoglycans (Table 2). Of the proteins in the ‘other’ category, nearly all were adhesive glycoproteins; these are linker proteins that connect cells with fibrous ECM. Of the collagens, the MS revealed that while α1 chain collagen VI (COL6A1) and α3 chain COL6 (COL6A3) were present in all ECMs derived from all cell types, α2 chain COL1 (COL1A2) was only in the DPfi and Rfi matrices, whereas α2 chain COLVI (COL6A2) and α1 chain COL1 (COL1A1) were only in the DPfi ECM. Of the glycoproteins, tenascin (TNC) and fibulin 2 were present in all ECMs, whereas THBS1 was present at high levels in the DPfi ECM, low levels in the Rfi ECM and was absent from the Pfi ECM. Vitronectin was present in the both the DPfi and Pfi ECM, whereas fibrillin 1 (FBN1) and emilin 1 were only found in the Rfi ECM (Fig. 3a). As noted above, we did not find any proteoglycans when the accuracy threshold was set to 95%. However, we did identify one proteoglycan – biglycan (BGN) – in the DPfi matrix at low levels when the accuracy threshold was lowered to 50%. We cannot be sure if other proteoglycans were expressed within our matrices as no other proteoglycans were detected with our technique. It is highly possible that the selected ECM extraction method selectively obtained only fibrous ECM and adhesive glycoproteins anchored to it.

In order to verify that proteins detected in our MS analysis were expressed within our cell‐derived ECMS, we stained each cell‐assembled ECM with antibodies against COL1 (all chains), COL6 (all chains), TNC and THBS1. Despite not finding COL1A1 or COL1A2 in our Pfi ECM by MS we found COL1 in all samples (Fig. 3b). The COL6 antibody we used also detects all α chains, and it appeared to be slightly more, but not significantly, abundant in the Pfi ECM, which correlates with observations from an earlier study (Fig. 3c).^55^ Likewise, TNC was present at similar levels across all cell‐derived ECMs (Fig. 3d). THBS1 was predominantly expressed in DPfi‐derived matrix (Fig. 3e), which corroborates our MS results. We also used image analysis to quantify expression levels, and confirm these observations (Fig. 3f–i). These results highlight that cells *in vitro* deposit matrices similar to the ECM they produce *in vivo*. For example, THBS1 expression is higher within the dermal papilla than the interfollicular skin dermis.^16^

## Discussion

In the last two decades the role of ECM in cell biology has become evident. Indeed, ECM is known to regulate cell behaviour, and it plays an essential role in organ development, function and repair.^62^, ^63^ On this basis, ECM as a molecular scaffold is fundamental for tissue homeostasis, and alterations in a specific ECM component can lead to disruption of this process.^64^ Physical properties such as topography and porosity of ECM can influence various anchorage‐related biological functions, such as cell division and migration.^65^

Given the importance of ECM in cell biology, ECM scaffolds are becoming commonplace for tissue repair.^45^ However, within the skin at least, dermal scaffolds used to promote regeneration are insufficient. They are composed of only a simple ECM, or decellularized reticular dermis, and they do not take into account the complexity of heterogeneity within whole‐skin dermis.^1^ These limitations highlight the need for bioinspired scaffolds for skin repair.

**Figure 4:**
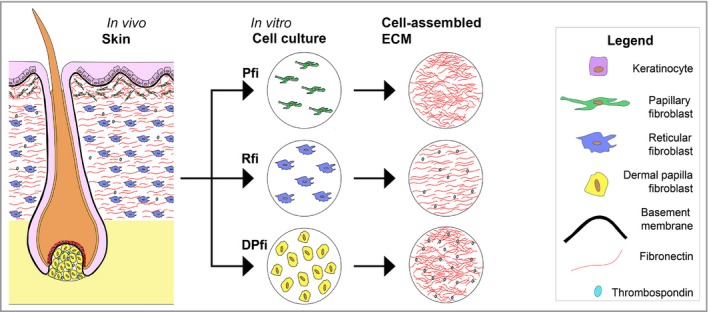
Summary of the results. Human skin *in vivo* has distinct features established by the fibroblast subtypes within the dermis that create and deposit extracellular matrix (ECM). For example, the dermal papilla is rich in thrombospondin, whereas the reticular fibroblast (Rfi)‐deposited matrix contains aligned ECM fibres. Fibroblasts isolated from the skin maintain distinct identities reflecting their origin, and subsequently deposit ECM *in vitro*, which reflects the location from which they were initially isolated. Pfi, papillary fibroblasts; DPfi, dermal papilla fibroblasts.

ECM scaffolds derived from cultured cells offer several advantages over decellularized tissues; there are lower risks of pathogen transmission and undesirable inflammatory and immunological reactions.^34^ In this study we showed that ECMs derived from three distinct fibroblast subtypes located within the skin dermis were morphologically, functionally and compositionally distinct from one another. In particular, in our study PFI were observed to generate matrices with significantly thicker fibers than DPfi and Rfi. This is different to how dermal ECM is organized in vivo, where the papillary dermis contains thinner fibers than the reticular dermis.^4^ However, with regard to organization we observed parallels in our cell‐assembled ECMs to ECM in different subanatomical locations from which the fibroblast subtypes were initially isolated. Specifically, the Pfi‐assembled matrix had significantly anisotropic fibres, which is analogous to the organization of the papillary dermis. The composition of cell‐assembled ECMs *in vitro* is also similar to the ECM in the spatial locations from where the cells were initially derived, indicating that fibroblasts *in vitro* have a memory of their subanatomical origin (Fig. 4). In particular, THBS1 was preferentially deposited by DPfi, whereas the *in vivo* dermal papilla is rich in THBS1. In addition, it is worth mentioning the absence of elastin and proteoglycans, which are usually key components of dermal ECMs.^66^ While we did detect BGN in our DPfi matrices, this was only at low levels when the accuracy threshold was lowered to 50%. However, previous studies of cell‐assembled ECM (by G.T. and J.T.C.) from dermal fibroblasts have identified proteoglycans, including versican and decorin, using antibodies for detection.^55^ We therefore believe the lack of proteoglycans is an artefact of matrix processing for MS. With regard to the lack of elastin in our matrices, we have an alternative explanation. In development, elastin is deposited relatively late, onto a scaffold of FBN1 microfibrils, which forms at an earlier time point.^67^ It may be the case that elastin was just not deposited in the 10‐day time frame over which ECMs were generated. In support of this, matrisome analysis of cell‐assembled matrices derived from neonatal dermal fibroblasts indicates that they also lack elastin.^53^ Despite this, fibrillins were observed in both our Rfi‐assembled matrices and the neonatal dermal fibroblast matrices, which act as a guide for elastin fibre deposition.

It is well known that the skin dermis and its interaction and crosstalk with epithelial cells are important for the production of the basement membrane components.^17^ In our study of epithelial‐only constructs, COL4 and COL7 were only found in keratinocytes of Pfi and DPfi ECM‐supported constructs, but not keratinocytes on Rfi ECM or control constructs. This observation is supported by previous studies, where authors have observed that decellularized fibroblast ECM stimulated the production and deposition of COL7 by keratinocytes.^61^ Refocusing on constructs supported by Rfi matrices, here we found no evidence of basement membrane establishment with either antibody staining or TEM. This is despite basement membrane formation occurring within our control samples and suggests that there may be an inhibitor of membrane formation within the Rfi matrices. While we removed cells from our matrices, growth factors may have remained attached to the ECMs onto which keratinocytes were seeded. We therefore cannot conclude that this observation is due specifically to the different ECMs, and it is highly likely that the growth factor constituents produced by the different fibroblast subtypes play an important role in directing the establishment of a basement membrane.^68^ Other studies have shown that when Pfi and Rfi are grown in co‐cultures with keratinocytes, they form cysts.^4^ In Rfi–keratinocyte cysts COL7 is absent; however, Pfi–keratinocyte cysts express all basement membrane components evaluated.^4^ Based on our observation and the aforementioned study, we postulate that basement membrane formation cannot be supported by ECM produced by Rfi. Again, this reflects the dermal locations of the cells *in vivo*, where supporting establishment of a basement membrane is not a usual Rfi cell function. In contrast, DPfi support establishment of a very thick basement membrane, reflecting their location subjacent to a thickened basement membrane, termed a glassy membrane *in vivo*.^14^ It is postulated that the expression of a high number of basement membrane ECMs in the dermal papilla, as opposed to the interfollicular dermis, means they have a key role in directing follicular physiology.^15^

In conclusion, the differences we observed in ECM composition, fibre morphology and behaviour between the different cell‐derived ECMs reflects differences that are physiologically present between the papillary, reticular and hair follicle dermis within the skin. We believe that inspiration can be taken from these physiologically different cell‐derived ECMs to improve the design of reliable biomimetic materials with improved therapeutic potential for skin tissue engineering. Self‐assembled scaffolds may be useful for clinical application because they mimic natural ECM structurally and compositionally, yet they do not elicit an immunogenic response. The self‐assembly process can also be used to establish ECMs of different thicknesses and strengths.^49^ Decellularized papillary dermis is not usually used as a tissue engineered product as it is very thin compared with the reticular dermis. Moreover, dermal papillae, which are < 100 μm in diameter, have never, to our knowledge, been decellularized and used in tissue engineering; they are simply too small. Using a self‐assembly approach, matrices derived from both DPfi or Pfi cultured *in vitro* could be superior to *ex vivo* Rfi‐derived dermis, or engineered dermal templates, as an effective product for skin engineering.

## Supporting information


**Powerpoint S1.** Journal Club Slide Set.
