# Supplementary material for: Subpopulations of dermal skin fibroblasts secrete distinct extracellular matrix: implications for using skin substitutes in the clinic
Source: Br J Dermatol. 2018 May 16;179(2):381–93. doi: 10.1111/bjd.16255 (PMC6175479; doi:10.1111/bjd.16255)
Supplement: Supplementary file 1 — Powerpoint S1. Journal Club Slide Set. [file BJD-179-381-s001.pptx]

## Slide 1
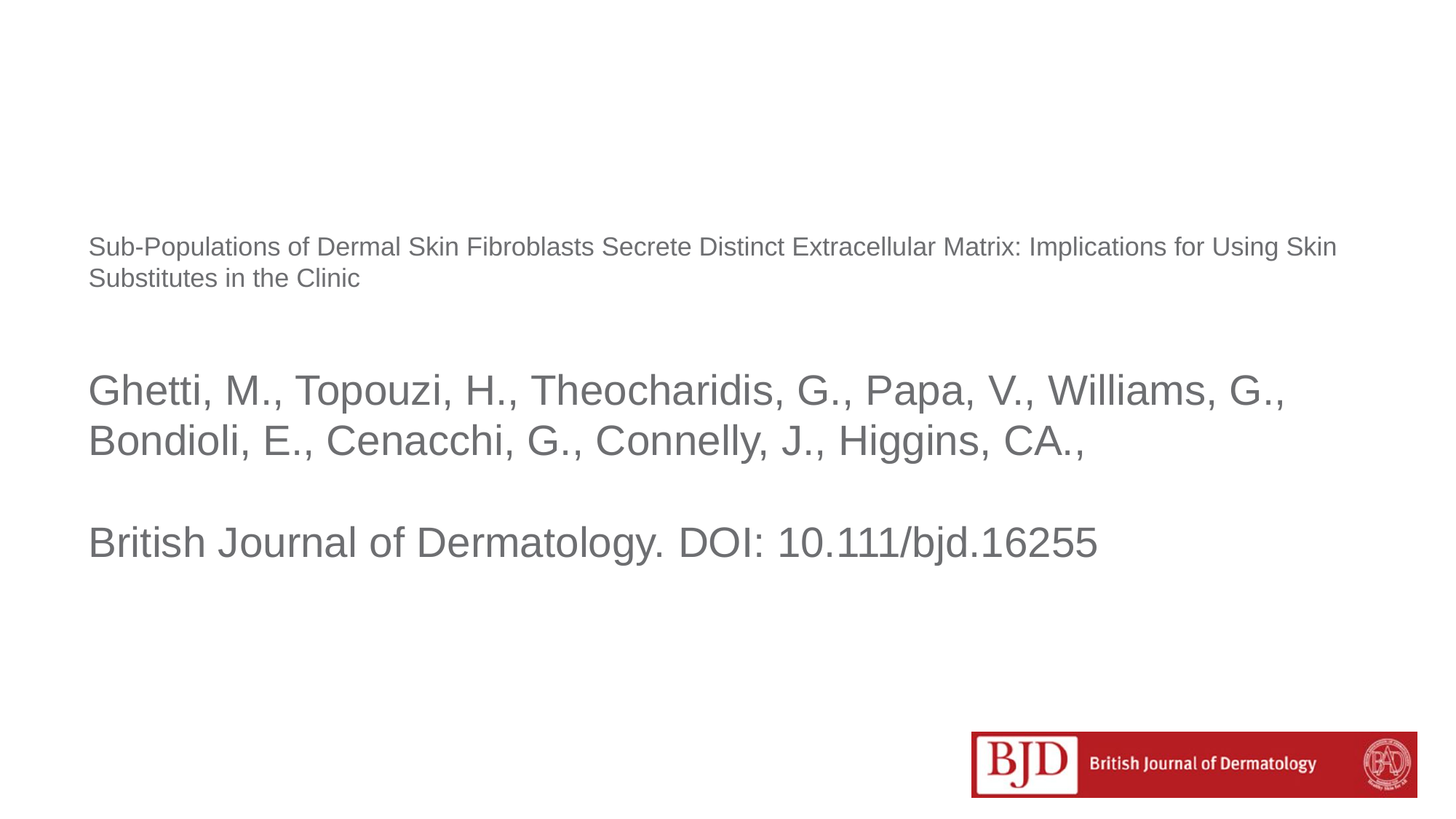

# Sub-Populations of Dermal Skin Fibroblasts Secrete Distinct Extracellular Matrix: Implications for Using Skin Substitutes in the Clinic
Ghetti, M., Topouzi, H., Theocharidis, G., Papa, V., Williams, G., Bondioli, E., Cenacchi, G., Connelly, J., Higgins, CA.,
British Journal of Dermatology. DOI: 10.111/bjd.16255

## Slide 2
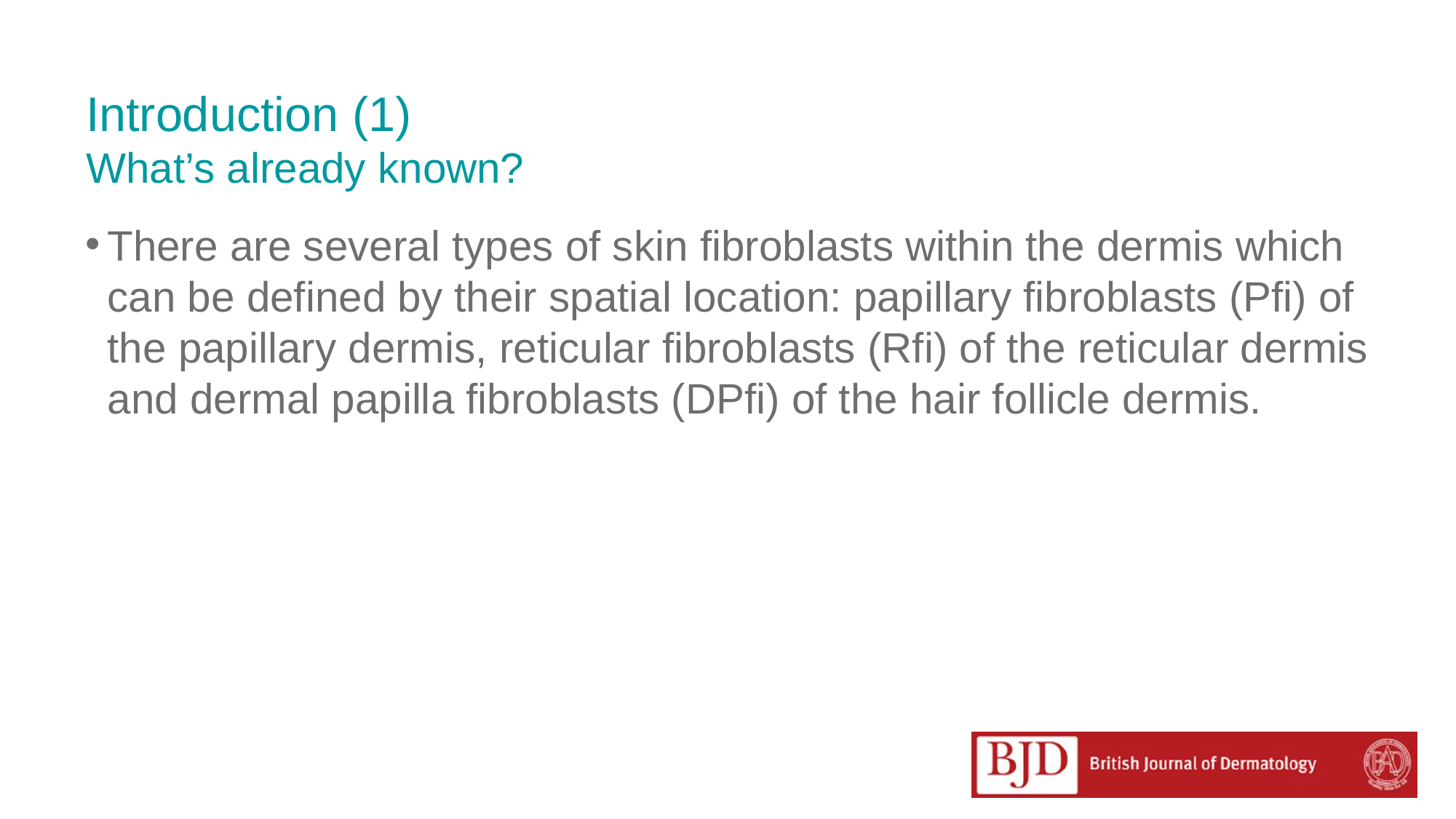

# Introduction (1) What’s already known?
There are several types of skin fibroblasts within the dermis which can be defined by their spatial location: papillary fibroblasts (Pfi) of the papillary dermis, reticular fibroblasts (Rfi) of the reticular dermis and dermal papilla fibroblasts (DPfi) of the hair follicle dermis.

## Slide 3
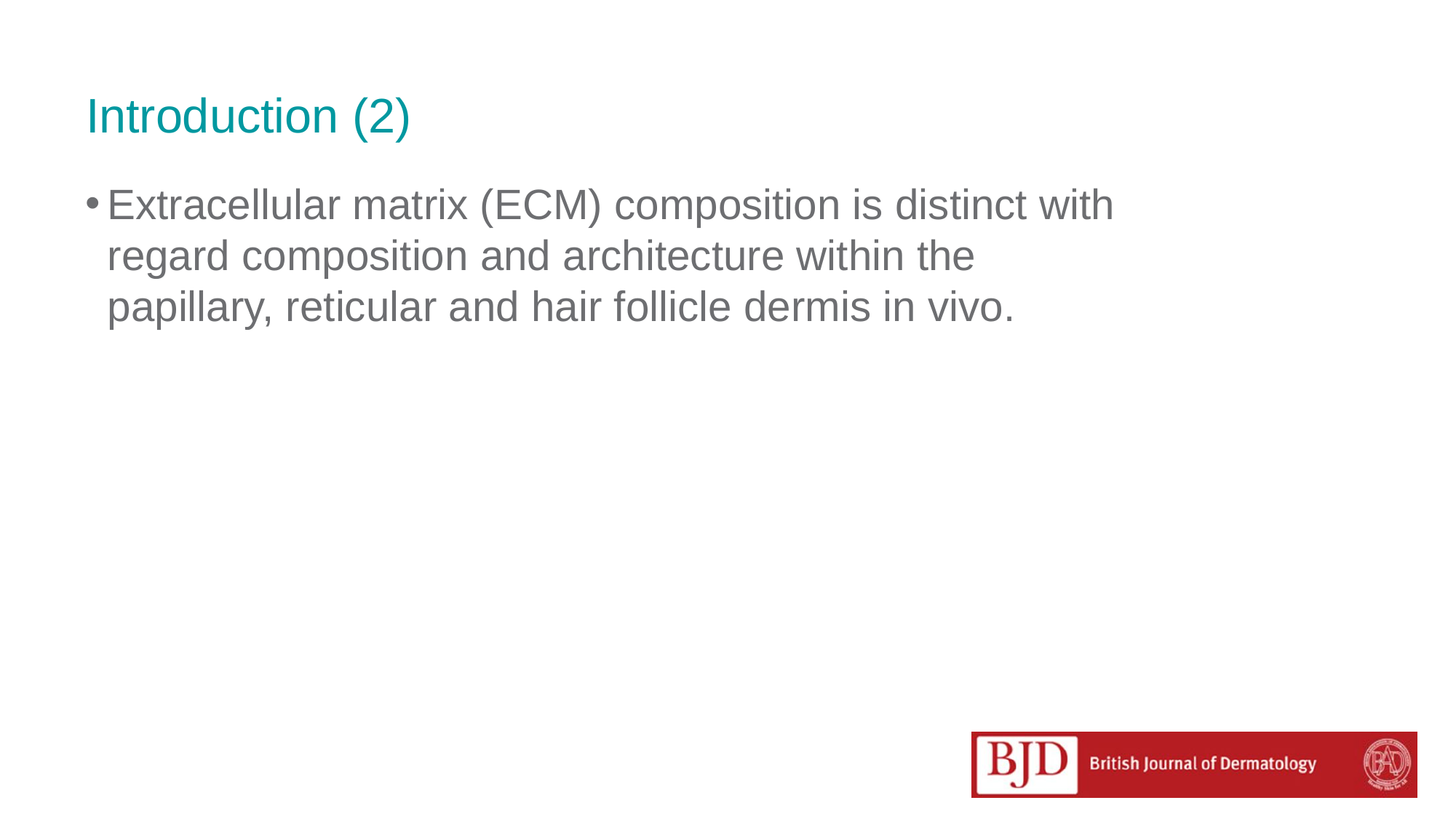

# Introduction (2)
Extracellular matrix (ECM) composition is distinct with regard composition and architecture within the papillary, reticular and hair follicle dermis in vivo.

## Slide 4
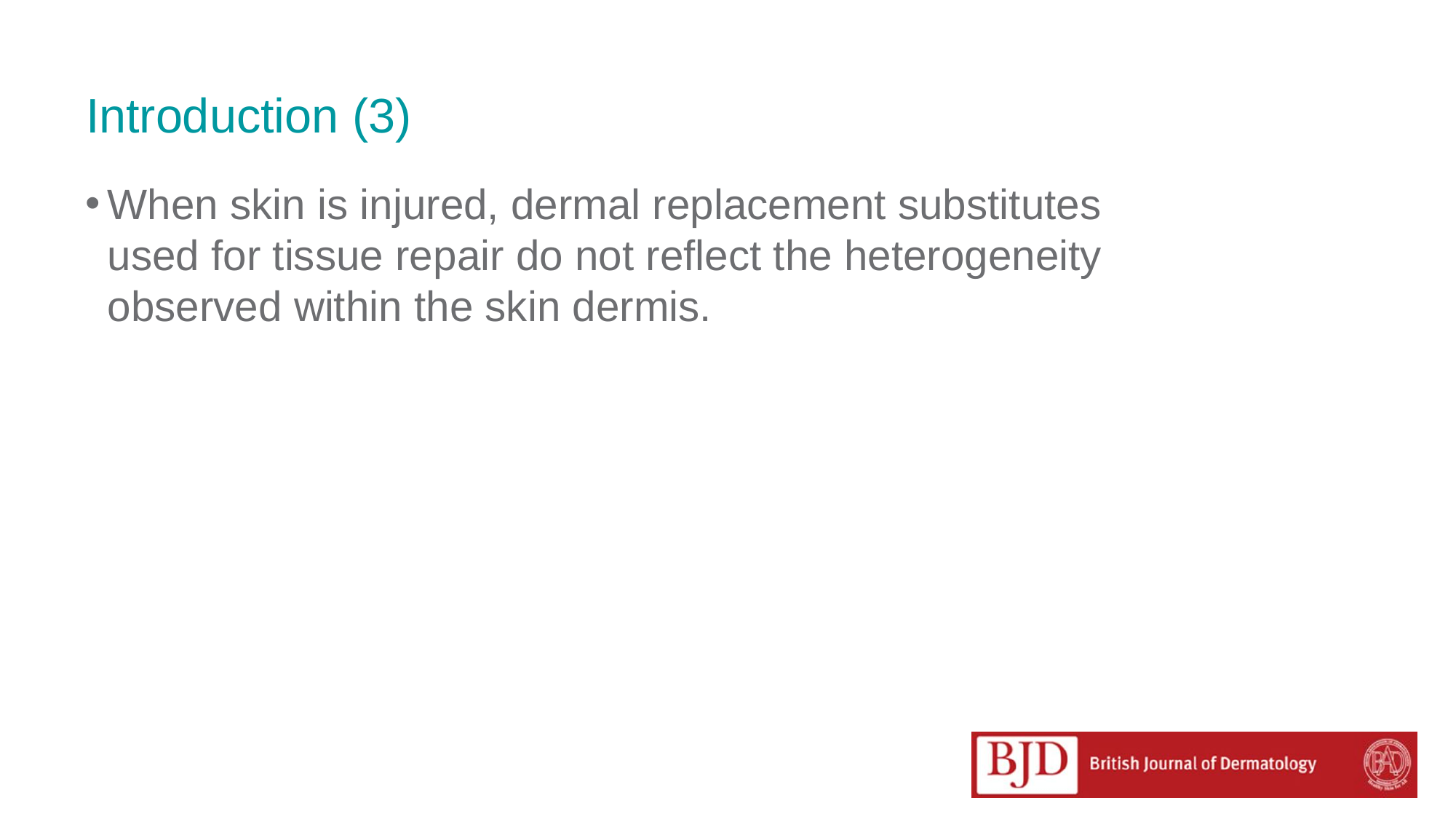

# Introduction (3)
When skin is injured, dermal replacement substitutes used for tissue repair do not reflect the heterogeneity observed within the skin dermis.

## Slide 5
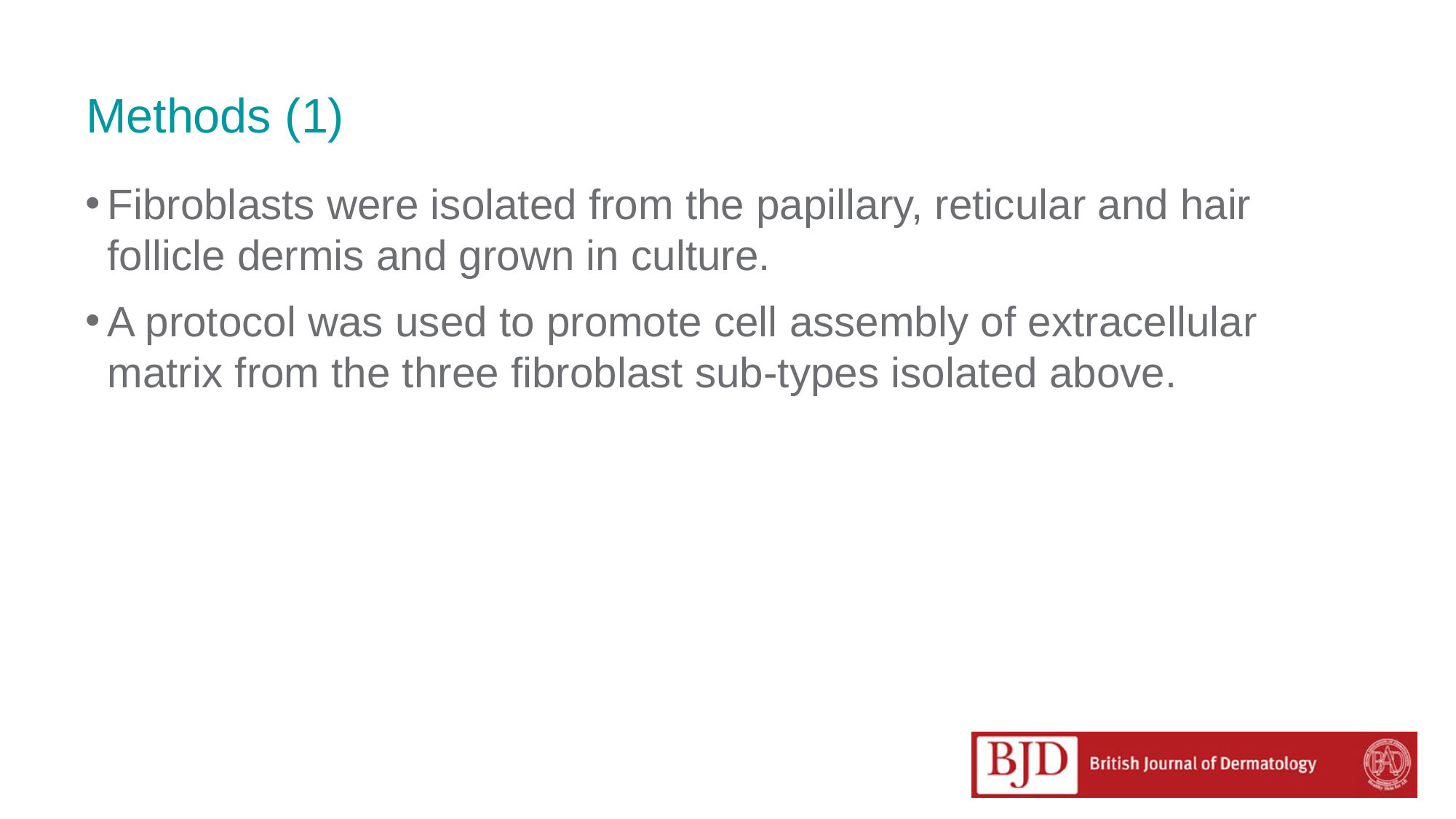

# Methods (1)
Fibroblasts were isolated from the papillary, reticular and hair follicle dermis and grown in culture.
A protocol was used to promote cell assembly of extracellular matrix from the three fibroblast sub-types isolated above.

## Slide 6
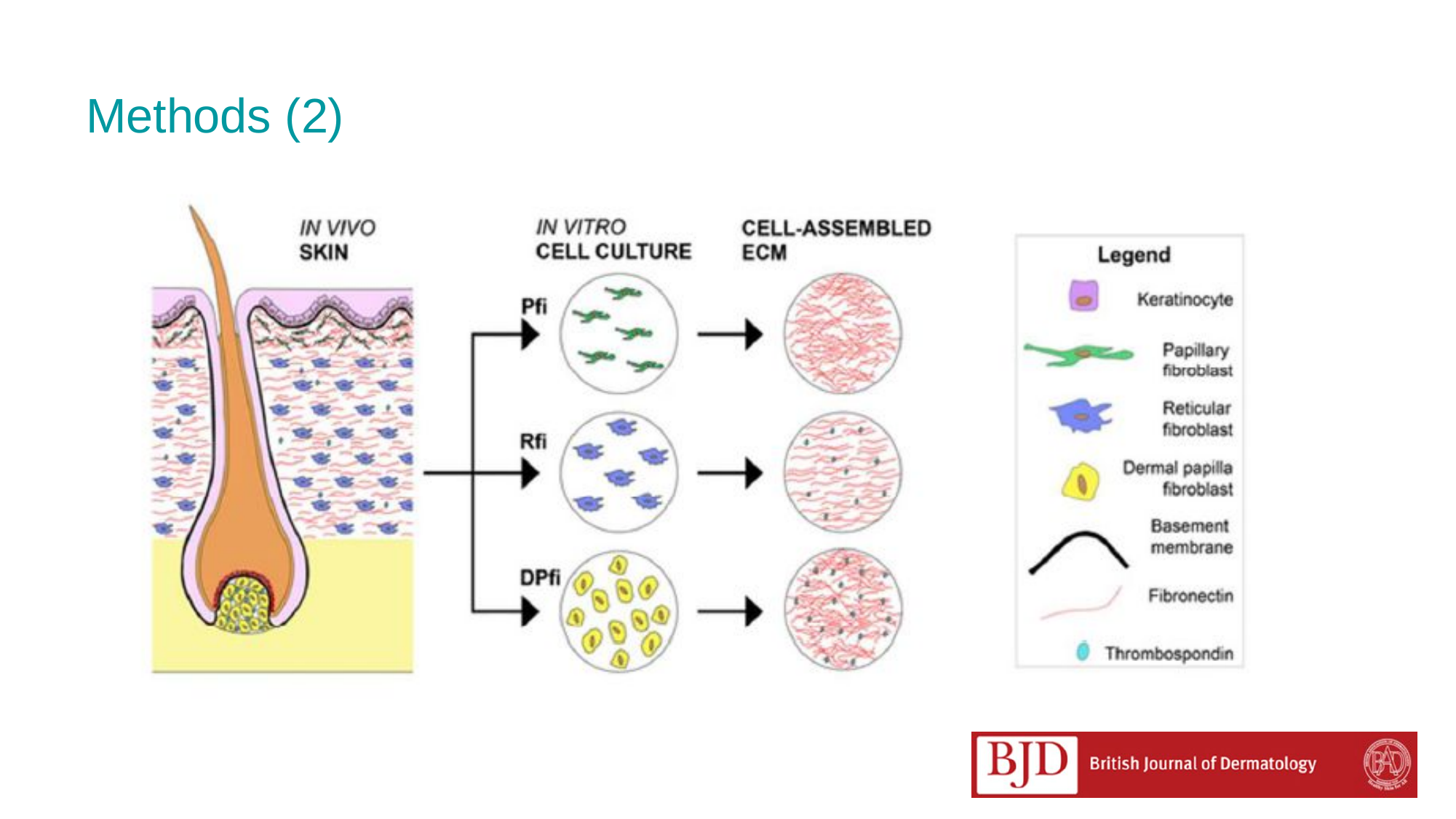

# Methods (2)

## Slide 7
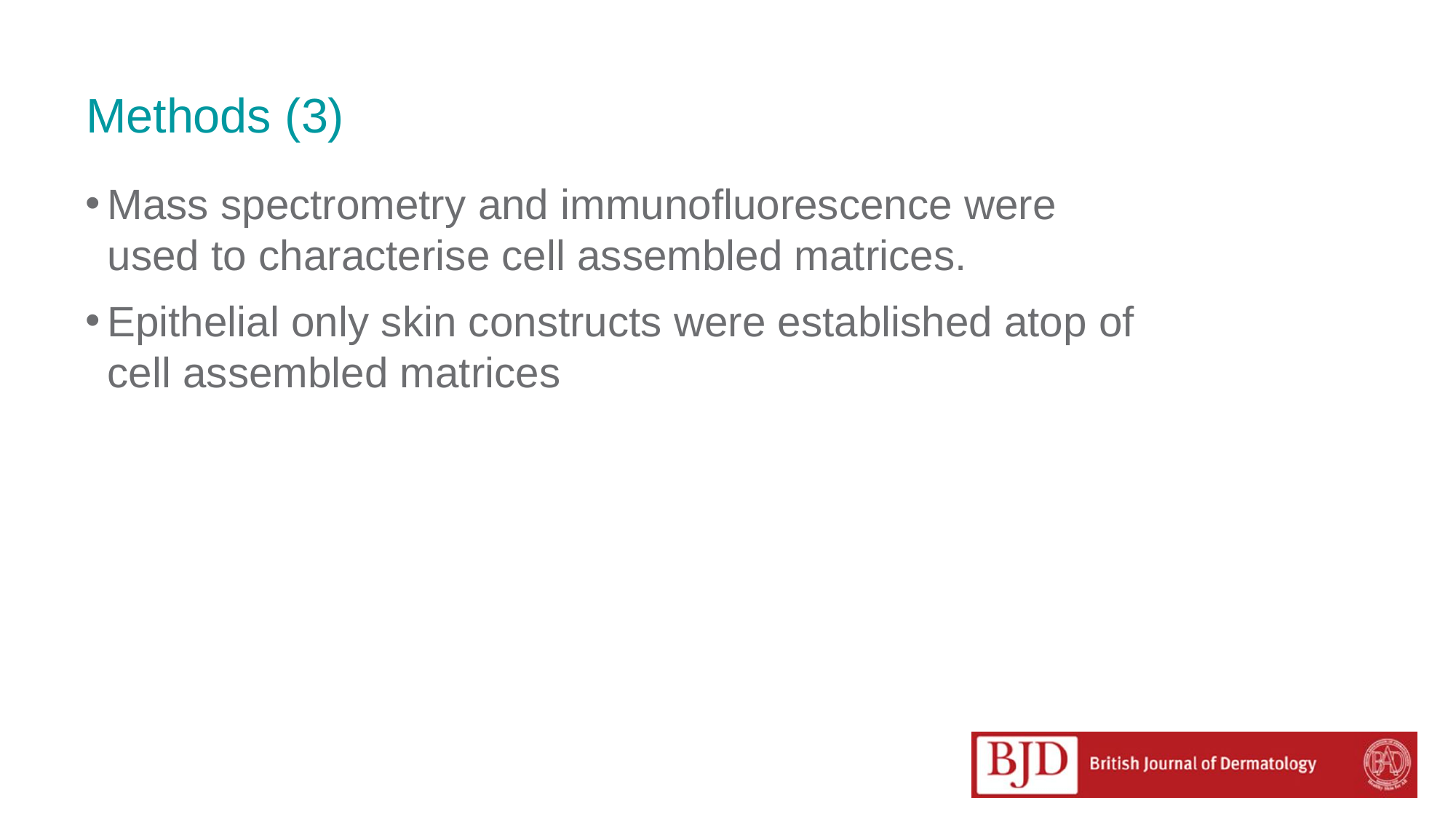

# Methods (3)
Mass spectrometry and immunofluorescence were used to characterise cell assembled matrices.
Epithelial only skin constructs were established atop of cell assembled matrices

## Slide 8
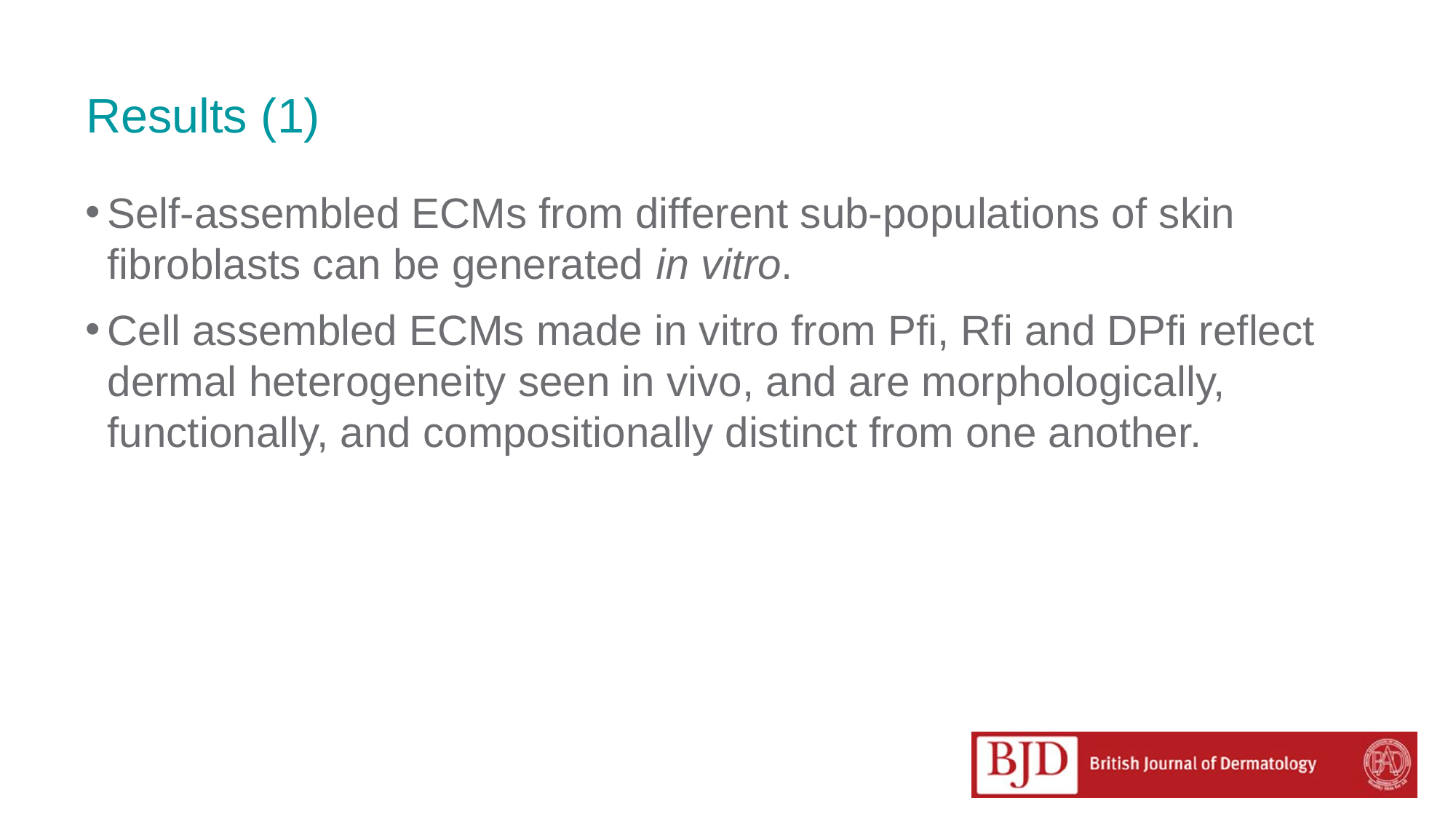

# Results (1)
Self-assembled ECMs from different sub-populations of skin fibroblasts can be generated in vitro.
Cell assembled ECMs made in vitro from Pfi, Rfi and DPfi reflect dermal heterogeneity seen in vivo, and are morphologically, functionally, and compositionally distinct from one another.

## Slide 9
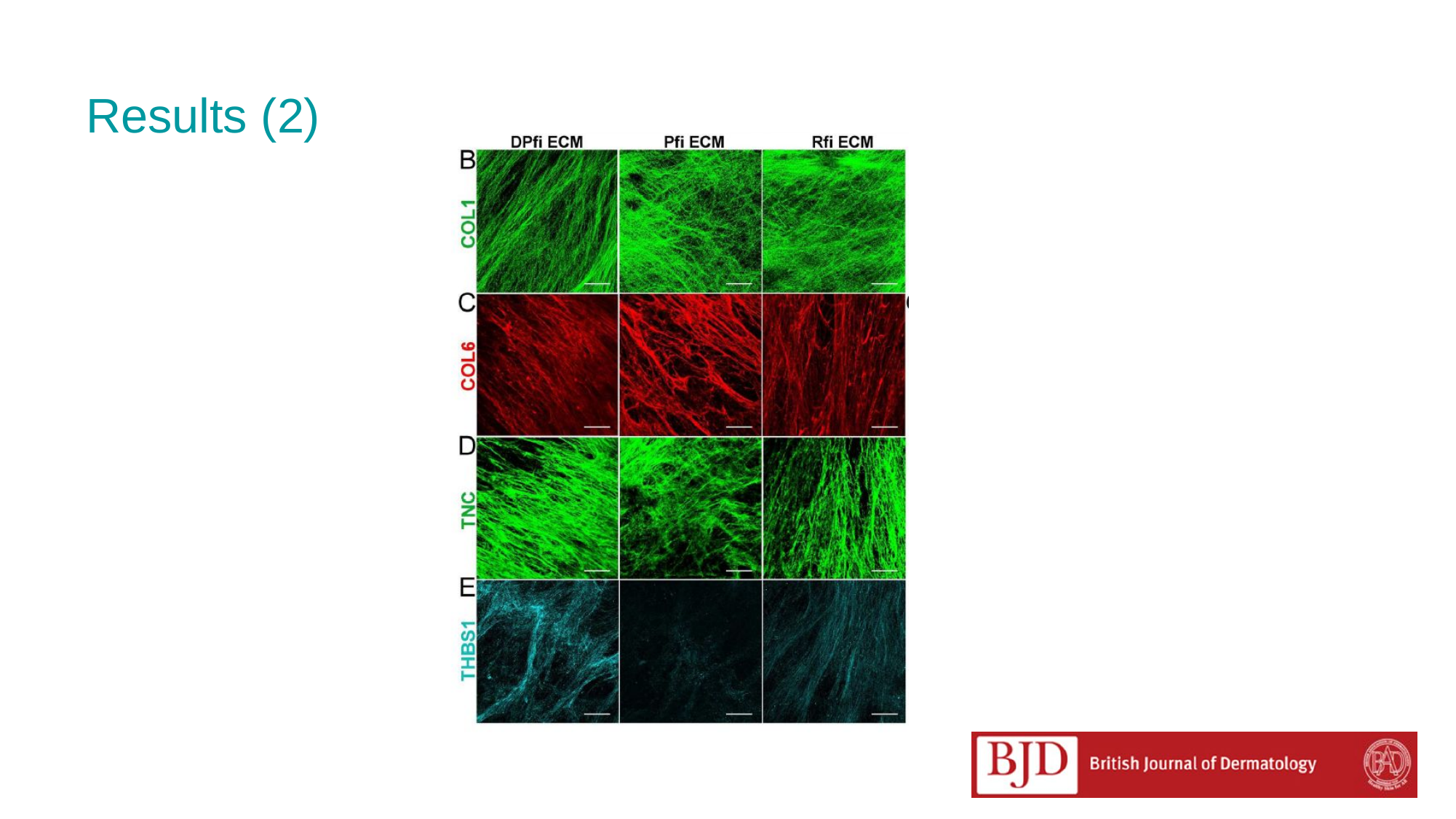

# Results (2)

## Slide 10
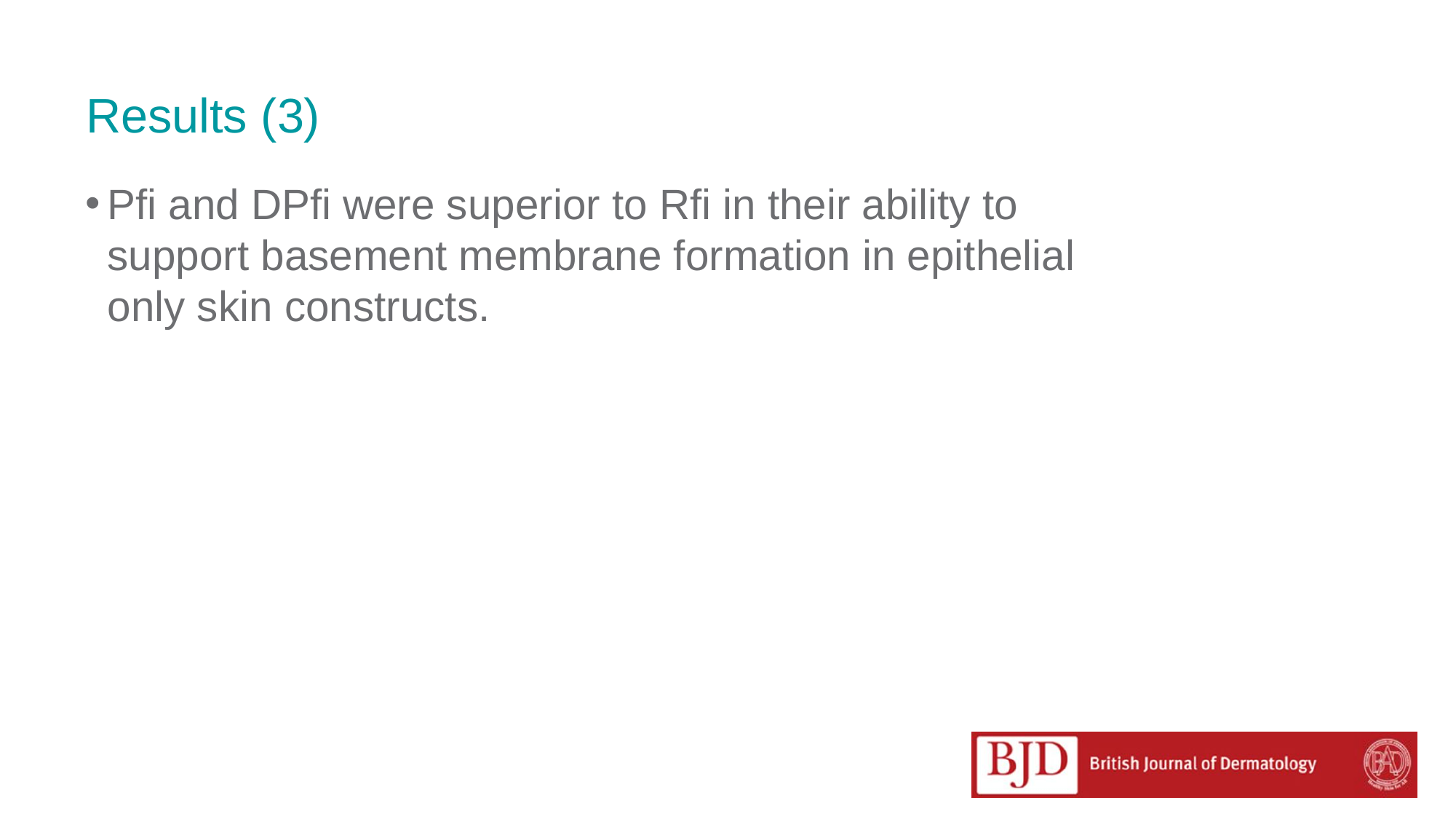

# Results (3)
Pfi and DPfi were superior to Rfi in their ability to support basement membrane formation in epithelial only skin constructs.

## Slide 11
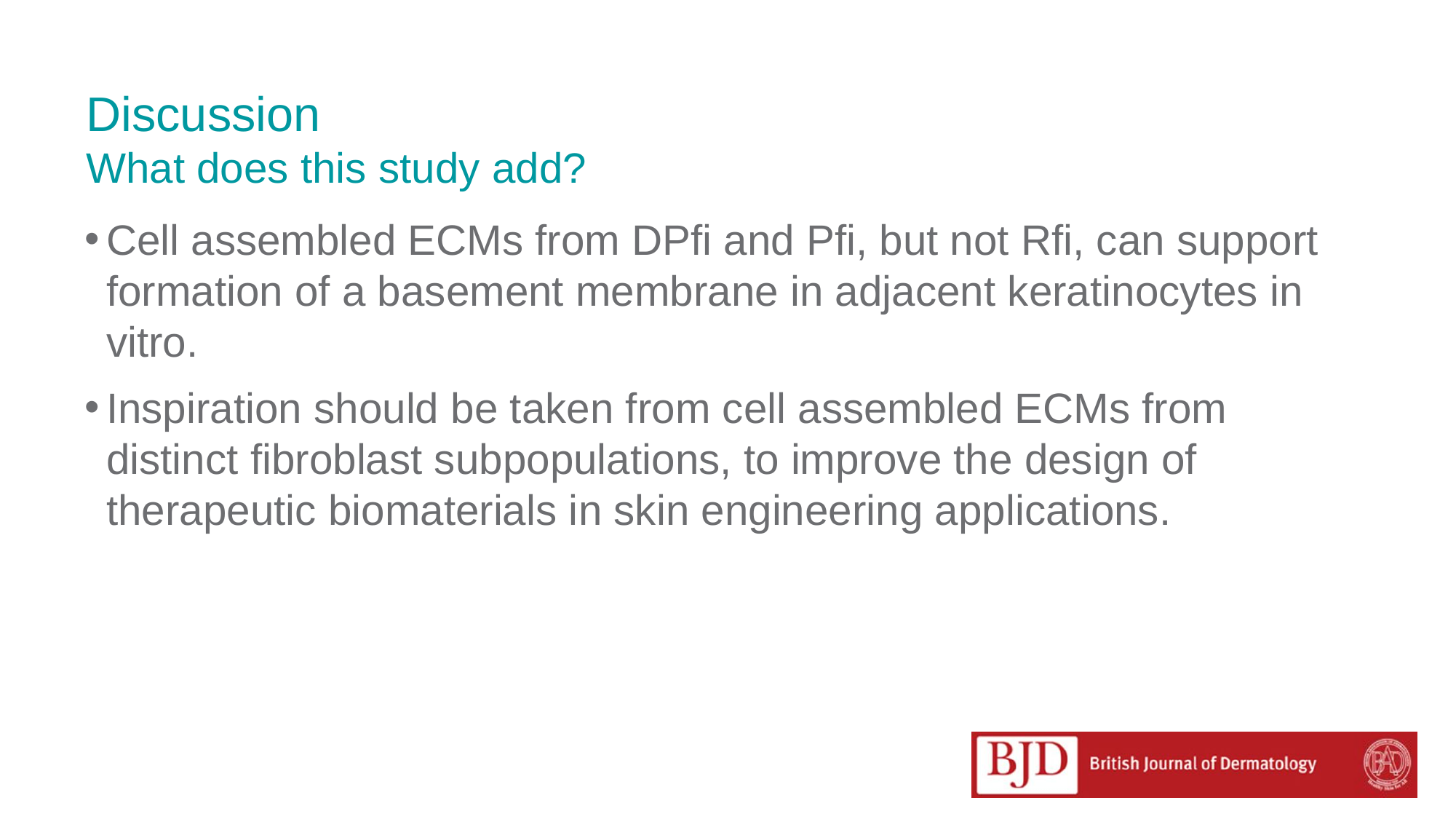

# Discussion What does this study add?
Cell assembled ECMs from DPfi and Pfi, but not Rfi, can support formation of a basement membrane in adjacent keratinocytes in vitro.
Inspiration should be taken from cell assembled ECMs from distinct fibroblast subpopulations, to improve the design of therapeutic biomaterials in skin engineering applications.

## Slide 12
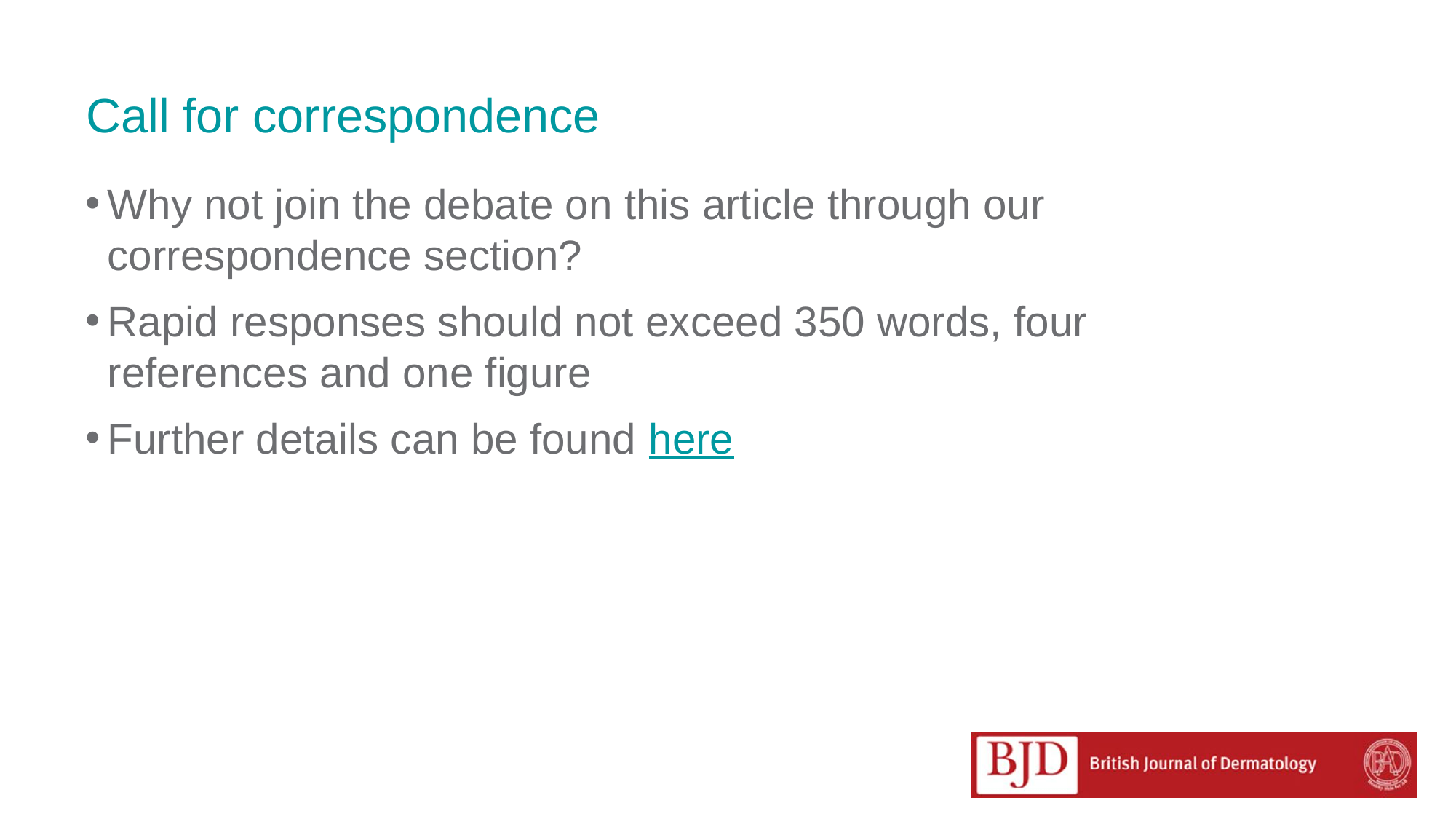

# Call for correspondence
Why not join the debate on this article through our correspondence section?
Rapid responses should not exceed 350 words, four references and one figure
Further details can be found here
